# First Evidence of Tris(catecholato)silicate Formation from Hydrolysis of an Alkyl Bis(catecholato)silicate

**DOI:** 10.3390/molecules27082521

**Published:** 2022-04-14

**Authors:** Vincenzo Campisciano, Benedetto Taormina, Alberto Spinella, Leonarda F. Liotta, Francesco Giacalone, Michelangelo Gruttadauria

**Affiliations:** 1 Department of Biological, Chemical and Pharmaceutical Sciences and Technologies (STEBICEF) and INSTM UdR—Palermo, University of Palermo, Viale delle Scienze, Building 17, 90128 Palermo, Italy; benedetto.taormina@you.unipa.it (B.T.); francesco.giacalone@unipa.it (F.G.); 2 ATeN Center, University of Palermo, Via F. Marini 14, 90128 Palermo, Italy; alberto.spinella@unipa.it; 3 Institute for the Study of Nanostructured Materials (ISMN), (Italian) National Research Council (CNR), Via Ugo La Malfa 153, 90146 Palermo, Italy; leonardafrancesca.liotta@cnr.it

**Keywords:** hybrid organic-inorganic material, hypervalent silicate, Knoevenagel reaction

## Abstract

The hydrolysis of 3-ammoniumpropylbis(catecholato)silicate **1,** giving two different silica-based materials containing different amounts of tris(catecholato)silicate, is reported. The latter species can be formed through an attack of catechol to the silicon atom in the pentacoordinate complex, in which the silicon-carbon bond is further activated toward electrophilic proton cleavage. The Knoevenagel reaction was used as a probe in order to test the availability of functional groups on the surface of such materials.

## 1. Introduction

The chemistry of catechol and silicon is very intriguing. As an example, the structure of the simple bis(catecholato)silane has been debated since 1951 [1], and only in 2021, a detailed discussion about its structural features has been reported [2]. Moreover, the chemistry of hypervalent penta- and hexacoordinated (catecholato)silicates is worthy of attention, both from a synthetic and biological standpoint, and still needs to be deeply investigated [3]. Pentacoordinate catecholate silicon complexes [4] have been employed for useful synthetic applications [5,6,7,8,9,10,11,12,13], for solid-phase synthesis [14], and for the preparation of porous pentacoordinate organosilicon frameworks [15] or for self-assembled macrocycles [16,17,18].

On the other hand, hexacoordinated catecholate silicon complexes have been used for reactions with nucleophiles [19,20], for anionic silicate organic frameworks [21], and for the construction of macrocycles [22,23,24] or three-dimensional polymeric networks [25], and in redox chemistry [26].

In addition, it was demonstrated that the existence of an organosilicon complex containing hexavalent silicon coordinated to at least one nitrogen formed during the life cycle of the diatom *Navicula pelliculosa* [27]. Stable, hypervalent silicon catecholate complexes have been implicated in the biosilicification process [28]. Si-enterobactin, a hexacoordinated silicon complex possessing a tris-catecholate moiety, was isolated from an endophytic *Streptomyces sp.* occurring in *Piper guinensis* roots. It is believed that such complex or related complexes may be involved in the transport of silicon in plants, diatoms, or other silicon-dependent organisms [29].

**Scheme 1:**
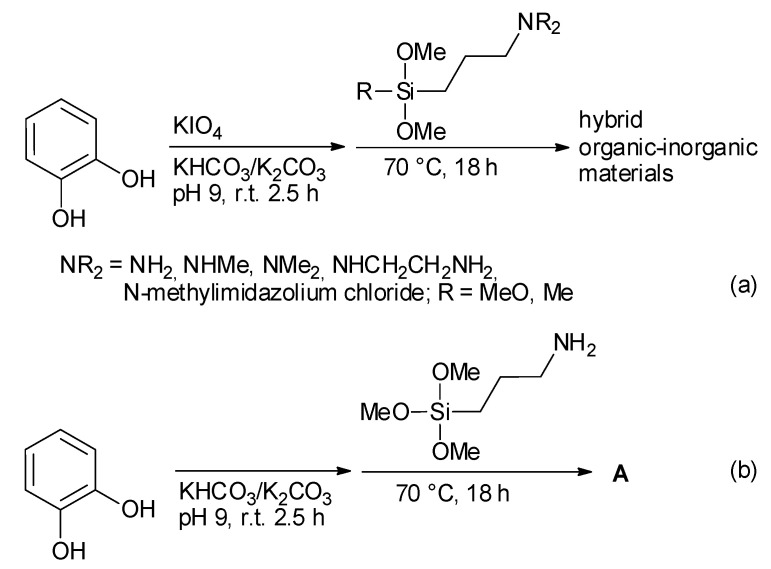
(**a**) General approach adopted for the synthesis of hybrid organic-inorganic materials with the use of KIO_4_ and (**b**) synthesis of material **A** without any oxidant.

Recently, we have described a simple one-pot procedure under mild conditions for the synthesis of a set of hybrid organic-inorganic multifunctional materials by mimicking polydopamine-like chemistry [30,31]. The synthetic procedure is based on the use of catechol and KIO_4_ as an oxidising agent, followed by the reaction with 3-aminopropyl-trimethoxysilane (or dimethoxymethyl-) or their corresponding substituted amines (Scheme 1a).

These materials were characterised by using several techniques, then, Knoevenagel reactions between ethyl cyanoacetate and several benzaldehydes were used as a probe in order to test the availability of functional groups on the surface of such materials.

**Figure 1:**
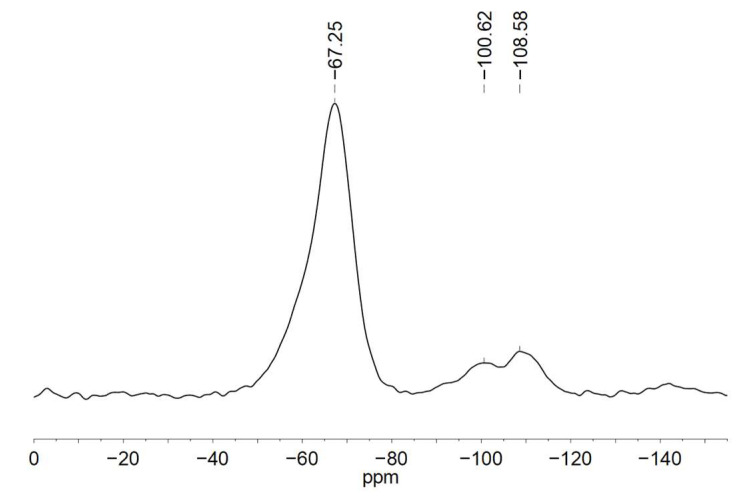
^29^Si CP-MAS NMR spectrum of material **A** (ref. [30]).

Among them, material **A** (Scheme 1b) had no catalytic effect on the reaction between ethyl cyanoacetate and 3-methoxybenzaldehyde, used as reaction test. In particular, material **A** had an inhibiting catalytic effect (6% yield ca.); indeed, the uncatalyzed reaction gave a higher yield (30%) when the reaction was carried out in ethanol [30]. This material was the only one that was synthesised without the use of the oxidant KIO_4_. Moreover, material **A** showed a higher content of aminopropyl loading, as determined by thermogravimetric analysis. The characterization of material **A** with ^29^Si CP-MAS NMR showed a peculiar aspect: different from the other materials, which exhibited only the presence of T signals, **A** also showed the presence of signals that could be ascribed to the Q system and a very small signal at ca. −140 ppm that could be due to hexavalent silicon species (Figure 1).

Therefore, in light of the above, the questions that arose were: why is material **A** not able to catalyse the Knoevenagel reaction though possessing the higher amine loading among the materials prepared? Is the presence of Q system signals in the ^29^Si CP-MAS NMR spectrum related to the low catalytic activity? How is the Q system originated? Is the very small signal at ca. −140 ppm related to hexacoordinated silicon species and, if correct, how it was formed? Does the understanding of this chemical behaviour shed some light on the development of new catalytic materials?

The answers to these questions through a deeper investigation of this material constitute the aim of this study.

## 2. Results and Discussion

**Scheme 2:**
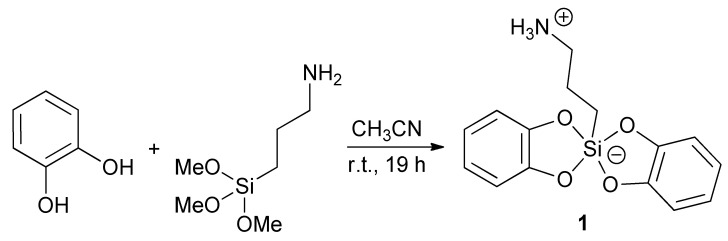
Synthesis of 3-ammoniumpropylbis(catecholato)silicate **1**.

As mentioned above, material **A** was prepared in the absence of KIO_4_; then, it was plausible that after the first step (see Scheme 1b) a very large amount of catechol was still present in the reaction mixture. The addition of 3-aminopropyl-trimethoxysilane may give a reaction with catechol to form a hypervalent pentacoordinated silicon compound that can further react to give the final material. With the aim to verify this hypothesis, we prepared 3-ammoniumpropyl-bis(catecholato) silicate **1** (^1^H and ^13^C NMR shown in Figures S1 and S2, ESI) by reaction of catechol and 3-aminopropyl-trimethoxysilane in acetonitrile (Scheme 2) [10].

**Scheme 3:**
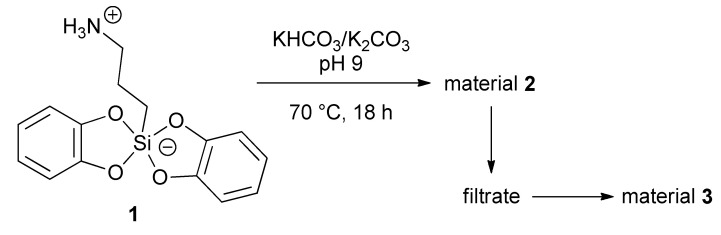
Synthesis of materials **2** and **3**.

With compound **1** in our hand, we carried out the reaction under the usual reaction condition for the preparation of the hybrid materials, i.e., KHCO_3_/K_2_CO_3_ at pH 9, 70 °C for 18 h (Scheme 3). After this time, we obtained a brown solid which was easily recovered by filtration (material **2**, Scheme 3). The filtrate was concentrated under reduced pressure, affording a sticky residue that was treated with methanol, giving a solid. The new precipitate was again recovered by filtration (material **3**, Scheme 3). This synthetic procedure was repeated another two times with identical results.

**Figure 2:**
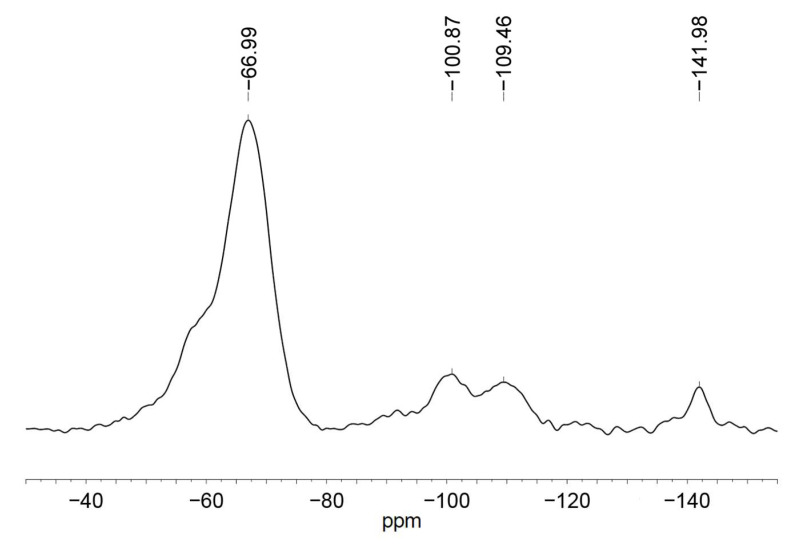
^29^Si CP-MAS NMR spectrum of material **2**.

Material **2** was characterised by means of ^13^C CP-MAS NMR (Figure S3, ESI) and ^29^Si CP-MAS NMR (Figure 2). The ^29^Si spectrum strictly resembles the one of material **A** (Figure 1). As a matter of the fact, in addition to the expected T signals (T^3^/T^2^ system), Q signals were present. In this case, the very small peak at ca. −140 ppm in material **A** was now clearly visible.

**Figure 3:**
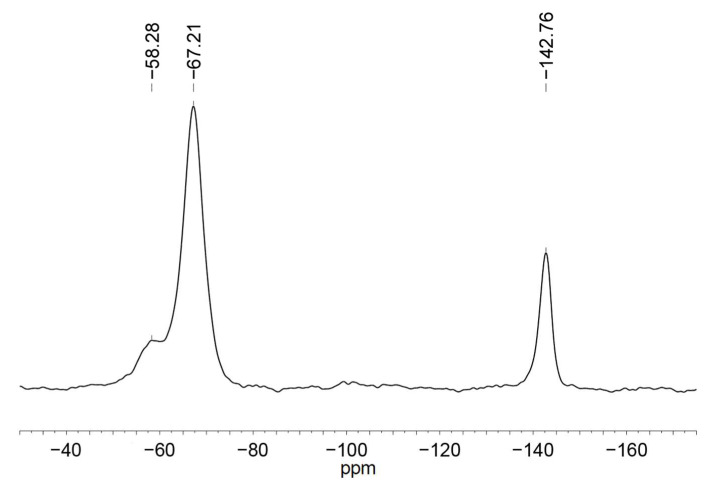
^29^Si CP-MAS NMR spectrum of material **3**.

Material **3** was characterised by means of ^13^C CP-MAS NMR (Figure S4, ESI) and ^29^Si CP-MAS NMR (Figure 3). ^13^C NMR of both materials exhibited signals due to the catecholate moiety as well as to the presence of the aminopropyl chain; in the case of material **3,** the catecholate moiety gave very sharp signals.

**Figure 4:**
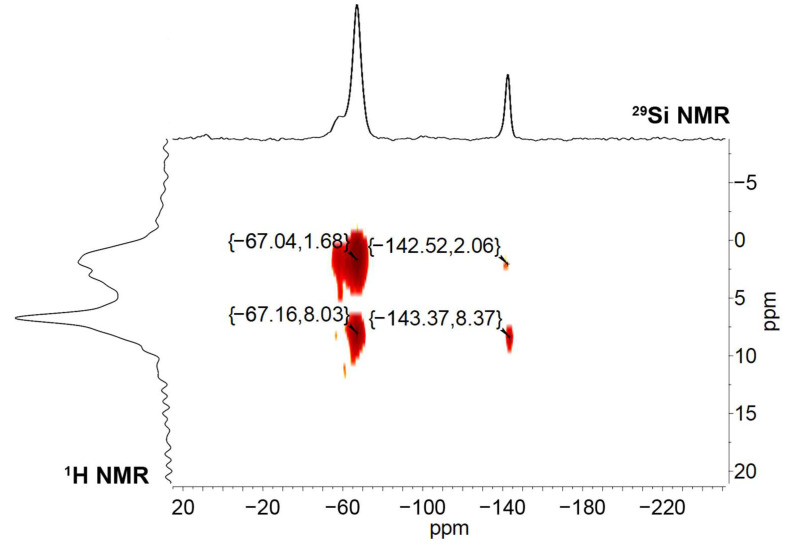
2D ^1^H/^29^Si FSLG/CP/MAS/HETCOR-NMR spectrum of material **3**.

The ^29^Si CP-MAS NMR spectrum of material **3** (Figure 3) showed a T^3^/T^2^ system and a strong signal due to the presence of hexavalent silicon species. No signals due to Q systems were present. In Figure 4, the 2D ^1^H/^29^Si FSLG/CP/MAS/HETCOR-NMR spectrum of material **3** is reported. From this spectrum, it is possible to observe a cross-peak between the propyl chain and the hexavalent silicon atom, which could indicate a close spatial relationship between them.

The thermogravimetric analysis of **2** and **3** is shown in Figure S5. The two materials appear to be thermally stable up to ca. 200 °C, whereas above this temperature, a weight loss due to the decomposition of the organic moieties can be observed. Material **3**, which contains a higher organic fraction, shows a faster decomposition compared to **2**, which is complete at ca. 600 °C, while the latter material is completely degraded at ca. 700 °C. Nitrogen physisorption analysis of materials **2** and **3** was carried out. Figures S6 and S7 show the reversible isotherms characterised by a sharp increase in the adsorbed volume at a relative pressure close to 1. The specific surface area (SSA) of the solids was calculated using the Brunauer–Emmett–Teller (BET) method (0.05 < P/P0 < 0.3). The solids display a low SSA (**2** 11 m^2^g^−1^; **3** 6 m^2^g^−1^).

As previously described, we again used the Knoevenagel reaction as a probe to test the availability of functional groups on the surface of such materials **2** and **3**. In Table 1, the results obtained in the reaction between ethyl cyanoacetate and 2,4-dimethoxybenzaldehyde are reported. First, we checked material **A**. In this case, a 17% conversion was observed (Table 1, entry 1). Material **2** gave a practically identical conversion (entry 2). On the other hand, material **3** showed a higher conversion (entry 3). Interestingly, the catalytic activity was even higher in the following two cycles (entries 4 and 5). However, in the fourth cycle, a consistent drop in the catalytic activity was observed (entry 6).

**Figure 5:**
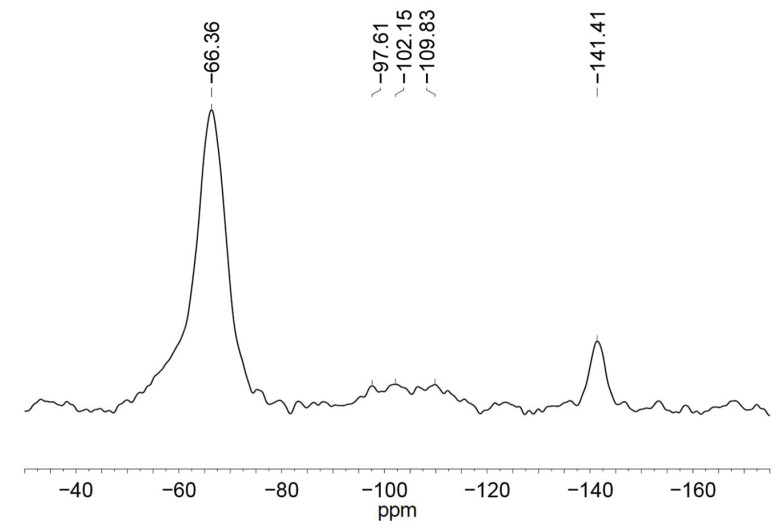
^29^Si CP-MAS NMR spectrum of material **r-3**.

In order to have an explanation of such behaviour, the ^29^Si CP-MAS NMR spectrum of the reused catalyst was carried out (Figure 5). Very interestingly, the spectrum of reused **3** (**r-3**) was similar to the spectrum of material **2** (see Figure 2 and Figure 5). As a matter of fact, signals related to the Q systems appeared, though with lower intensity with respect to material **2**, whereas the intensity of the signal belonging to the hexavalent silicon species was much lower.

At this point, some considerations can be proposed. Firstly, materials **2** and **A** possess similar features: high silica content (41.4% and 49.2%, respectively), very similar ^13^C and ^29^Si NMR spectra, and very similar catalytic activity. Then, the starting hypothesis that compound **1** had a role in the formation of material **A** seems plausible. Secondly, the presence of Q signals can be related to lower catalytic activity, whereas the presence of hexavalent silicon species is related to a higher catalytic activity.

**Scheme 4:**
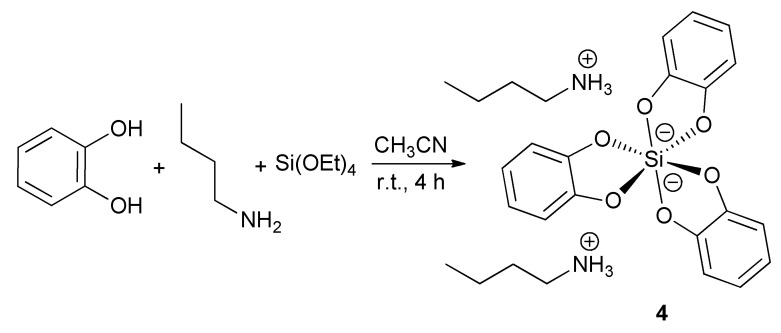
Synthesis of bis(butylammonium) tris(catecholato)silicate **4**.

In order to have deeper information about the role of hexavalent silicon species, we prepared the bis(butylammonium) tris(catecholato)silicate **4** (Scheme 4) [32,33], which was characterised by means of ^1^H and ^13^C NMR (Figures S8 and S9, ESI). Then, we used compound **4** in the Knoevenagel reaction (Table 1, entries 7 and 8). We found a complete conversion in the reaction between 2,4-dimethoxybenzaldehyde and ethyl cyanoacetate when catalyst **4** was used in ethanol in 0.33 mol% loading, whereas a 72% conversion was obtained when catalyst **4** was used in only 0.09 mol% loading.

These experiments confirmed the high catalytic activity of the hexavalent silicon species.

**Scheme 5:**
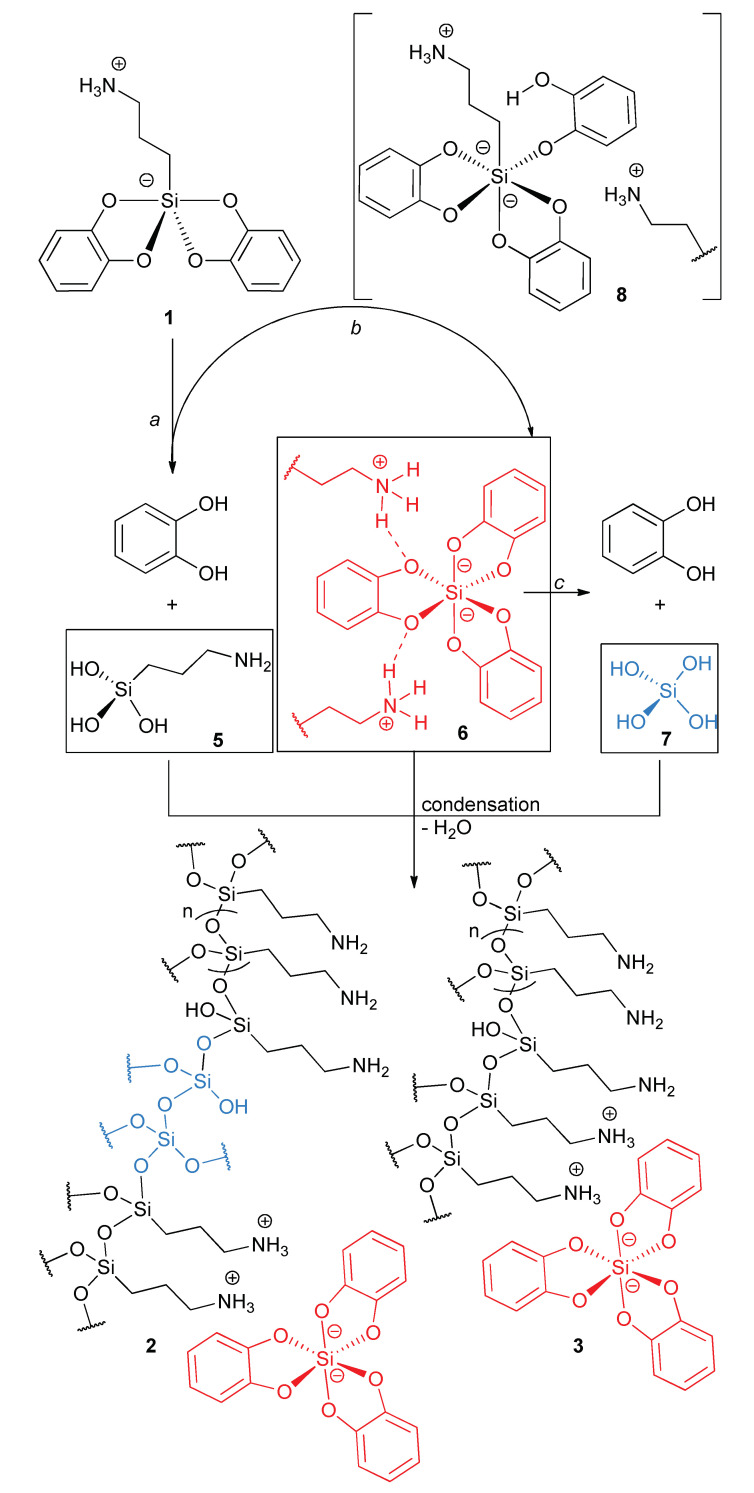
Proposed mechanistic pathways for materials **2** and **3**.

On the basis of this data, here we propose a possible mechanistic pathway. 3-Ammoniumpropylbis(catecholato) silicate **1** may start its hydrolysis process when heated at 70 °C in the buffer medium to give catechol and silane **5** (Scheme 5, pathway *a*). The catechol formed may react with unreacted compound **1** (pathway *b*). The silicon atom in the pentacoordinate complex **1** is still a Lewis acid, which is able to accept another Lewis base to form an intermediate hexacoordinate silicon complex **8**, in which the silicon–carbon bond is further activated toward electrophilic proton cleavage [34,35] to give the tris-catecholate hexavalent silicon species **6**. This is the crucial step. The Si–C bond cleavage reaction is a known reaction [4,36], even if, to the best of our knowledge, the transformation of a pentacoordinate silicon compound to a hexacoordinated silicon compound with the concomitant Si–C bond cleavage has not been described. Nevertheless, we should bear in mind that the reaction is carried out at 70 °C, not at room temperature, as for the known Si–C bond cleavage process. Then, the tris-catecholate hexavalent silicon **6** may undergo hydrolysis to give orthosilicic acid and catechol (pathway *c*) [37,38]. Indeed, silicon catecholate salt [39,40] has been used as silicic acid precursors to investigate silicification in vitro in the presence of amino acids or small peptide oligomers. Biomolecules such as polyamines [41,42] are present when silica is formed within an organism playing an important role in orthosilicic acid condensation. In our case, the presence of the protonated amine moieties could have played a role.

Condensation reaction first afforded material **2,** which is rich in aminopropyl moieties (T^3^/T^2^ systems) and poor in Q^4^/Q^3^ system (blue) and hexavalent silicon (red). Water removal under reduced pressure of the filtrate caused a further condensation reaction of the more polar fractions giving, after treatment of the sticky residue, material **3,** which is rich in aminopropyl moieties and hexavalent silicon. The large presence of the tris-catecholate silicon moiety in **3** is in agreement with the higher organic content observed in the TGA. Although there is a high content of aminopropyl moieties in material **2** (and in material **A**), the catalysis of the Knoevenagel reaction was modest, probably because the pure silica fraction was formed later in the condensation process, then covering the aminopropyl-based silica and causing partial unavailability of these functional groups. On the other hand, the high catalytic activity of **3** is due to the presence of the ammonium tris(catecholato)silicate moieties. The drop in the catalytic activity of material **3** after reuse could be due to the same reason: that it is the hydrolysis of the hexavalent silicon species to give silica that covered the aminopropyl functional groups. From these data, it is also deduced that material **A** has a similar structure to material **2**.

## 3. Materials and Methods

### 3.1. Spectroscopic and Analytical Methods

Chemicals and solvents were purchased from commercial suppliers to be used without further purification. Catechol (99%), 3-aminopropyl-trimethoxysilane (97%), tetraethyl orthosilicate (99%), and ethyl cyanoacetate (99%) were purchased from Fluorochem, whereas butylamine (99.5%), 2,4-dimethoxybenzaldehyde (98%), KHCO_3_ and K_2_CO_3_ were purchased from Sigma-Aldrich. All solvents (ACS grade) were purchased from VWR. Thermogravimetric analysis (TGA) measurements were carried out under oxygen flow from 100 to 1000 °C with a heating rate of 10 °C min^−1^ in a Mettler Toledo TGA STAR. Combustion chemical analysis was performed on a PerkinElmer (Waltham, MA, US) 2400 Series II Elemental Analyzer System. Nitrogen adsorption–desorption analysis was performed at 77 K by using a volumetric adsorption analyzer (Micromeritics (Norcross, GA, US) ASAP 2420). Before the analysis, the sample was pre-treated at 150 °C for 16 h under reduced pressure (0.1 mbar). The BET method was applied in the p/p_0_ = 0.05–0.30 range to calculate the specific surface area. ^1^H-^29^Si CP MAS spectra were obtained at room temperature by means of a Bruker (Billerica, MA, US) Avance II 400 MHz (9.4 T) spectrometer operating at 79.4 MHz for the ^29^Si nucleus with a MAS rate of 6kHz, 2048 scans, a delay time of 8s, and a contact time of 8 ms.

^1^H-^13^C CP MAS spectra were obtained at room temperature operating at 100.6 MHz for the ^13^C nucleus with a MAS rate of 6 kHz, 1000scans, a delay time of 4 s, and a contact time of 1.5 ms. ^1^H-^29^Si HETCOR experiments with Lee–Goldburg (LG) ^1^H homonuclear decoupling were performed by applying the FSLG decoupling during the t1 evolution period. An FSLG homonuclear decoupling field strength of 73.5 kHz, a contact time of 500 μs and a MAS rate of 10 kHz were used together with 1024 points for 128 experiments.

### 3.2. Synthesis of 3-Ammoniumpropylbis(catecholato)silicate ***1***

In a 50 mL round bottom flask was placed 1.101 g (9.9 mmol) of catechol in CH_3_CN (25 mL). Then, 3-aminopropyl-trimethoxysilane (0.89 mL, 4.96 mmol) was added. The reaction mixture was allowed to stand for 18 h. After this time, the precipitate was filtered under reduced pressure and washed with CH_3_CN (ca. 200 mL). The solid was dried in an oven at 60 °C. Isolated yield 99%. ^1^H NMR (300 MHz, DMSO-d_6_, δ): 6.66–6.36 (m, 8H), 2.61 (t, J = 7.5 Hz, 2H), 1.61–1.36 (m, 2H), 0.64–0.40 (m, 2H) ppm. ^13^C NMR (75 MHz, DMSO-d_6_, δ): 150.82, 117.78, 110.16, 42.42, 23.38, 15.38 ppm.

### 3.3. Synthesis of Materials ***2*** and ***3***

In a 250 mL round bottom flask was placed 1.5 g (5 mmol) of 3-ammoniumpropylbis(catecholato) silicate **1**. Then, 150 mL of buffer solution (KHCO_3_/K_2_CO_3_ 10 mM, pH 9) was added. The reaction mixture was vigorously stirred at 70 °C. After the dissolution of compound **1**, the solution turned dark red, and a precipitate started to form. After 18 h, the reaction mixture was filtered under reduced pressure and the solid was washed with distilled water, ethanol, ethyl acetate and diethyl ether. The obtained red material **2** was dried in an oven at 60 °C (330 mg). The filtrate (water and ethanol) was evaporated under reduced pressure to give a brown sticky residue that was treated with methanol (150 mL). The mixture was stirred at room temperature to give a slightly red precipitate, which was filtered under reduced pressure. The solid was washed with methanol and diethyl ether, then dried in an oven at 60 °C to give material **3** (290 mg).

### 3.4. Synthesis of Dibutylammonium Tris(catecholato)silicate ***4***

In a 100 mL round bottom flask was placed 3 g (27 mmol) of catechol in CH_3_CN (25 mL). Then, butylamine (1.8 mL, 18 mmol) is added dropwise. A slightly turbid-pale red solution was obtained. To this solution, tetraethyl orthosilicate (2.03 mL, 9 mmol) was added dropwise for 30 min. At the end of the addition, a white suspension was formed, and the reaction mixture was left to stir at room temperature for 4 h. After this time, the reaction mixture was filtered under reduced pressure and the solid was washed with CH_3_CN (ca. 300 mL). The solid was dried in an oven at 60 °C. Isolated yield 82%. ^1^H NMR (300 MHz, DMSO-d_6_, δ) 6.59–5.90 (m, 12H), 3.13–2.61 (m, 4H), 1.52 (m, 4H), 1.40–1.05 (m, 4H), 0.85 (t, J = 7.3 Hz, 6H) ppm. ^13^C NMR (75 MHz, DMSO-d_6_, δ): 152.32, 115.77, 109.43, 29.60, 19.69, 13.98 ppm.

### 3.5. General Procedure for the Knoevenagel Reaction

In a vial, we placed 2,4-dimethoxybenzaldehyde (2 mmol), ethyl cyanoacetate (2 mmol) EtOH (1 mL), and catalyst (20 mg), and the reaction mixture was stirred at 50 °C for 2 h. To recover the catalytic material, the following procedure was applied: a mixture of CH_2_Cl_2_/Et_2_O 4:1 was added to the vial and the mixture was sonicated for a few seconds. Then, the mixture was centrifuged for 10 min. The supernatant liquid was removed, leaving the catalyst at the bottom of the vial. This procedure was repeated another five times. The catalyst was dried in the vial at 60 °C and was used for the next run. In the case of catalyst **4,** two reactions were carried out by using, respectively, 0.33 mol% and 0.09 mol% at 50 °C for 0.5 h.

## 4. Conclusions

In conclusion, we have described the hydrolysis of 3-ammoniumpropylbis(catecholato)silicate **1,** giving two different silica-based materials containing different amounts of the tris(catecholato)silicate moiety. The latter moiety is formed via the transformation of the pentacoordinated silicon compound to hexacoordinated silicon compound with the concomitant Si-C bond cleavage. As far as we know, this transformation has not been described before and, given the importance of silica-based organic–inorganic hybrid materials, it could add further knowledge to silicon chemistry.

## Figures and Tables

**Table 1:**
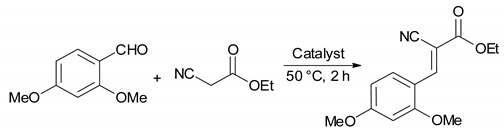
Knoevenagel reaction catalysed by materials **A**, **2**, **3** ^1^ and compound **4** ^2^.

Entry	Cycle	Catalyst	Conv.%
1	1	**A**	17
2	1	**2**	18
3	1	**3**	80
4	2	**3**	>99
5	3	**3**	>99
6	4	**3**	44
7	-	**4** (0.33 mol%)	>99
8	-	**4** (0.09 mol%)	72

^1^ Reaction condition: 2,4-dimethoxybenzaldehyde (2 mmol), ethyl cyanoacetate (2 mmol) EtOH (1 mL), catalyst (20 mg), 50 °C, 2 h. ^2^ reaction time: 0.5 h.

## Data Availability

Not applicable.

## Supplementary Materials

The following supporting information can be downloaded at: https://www.mdpi.com/article/10.3390/molecules27082521/s1, Structure of compounds and materials **1–4**. Figure S1: ^1^H NMR (300 MHz, DMSO-d6) of compound **1**; Figure S2: ^13^C NMR (75 MHz, DMSO-d6) of compound **1**; Figure S3: ^13^C CP-MAS NMR of material **2**; Figure S4: ^13^C CP-MAS NMR of material **3**; Figure S5: TGA graph of materials **2** and **3**; Figure S6: N_2_-adsorption/desorption isotherms of material **2**; Figure S7: N_2_-adsorption/desorption isotherms of material **3**; Figure S8: ^1^H NMR (300 MHz, DMSO-d6) of compound **4**; Figure S9: ^13^C NMR (75 MHz, DMSO-d6) of compound **4**.

## Sample Availability

Samples of the compounds **A**, **1**, **2**, **3**, and **4** are available from the authors.
