# Supplementary material for: First Evidence of Tris(catecholato)silicate Formation from Hydrolysis of an Alkyl Bis(catecholato)silicate
Source: Molecules. 2022 Apr 14;27(8):2521. doi: 10.3390/molecules27082521 (PMC9032887; doi:10.3390/molecules27082521)
Supplement: Supplementary file 1 [file molecules-27-02521-s001.zip › molecules-1658633-supplementary.pdf]

SUPPORTING INFORMATION

First evidence of tris(catecholato)silicate formation from hydrolysis of an alkyl bis(catecholato)silicate

Vincenzo Campisciano [1,*], Benedetto Taormina [1], Alberto Spinella [2], Leonarda F. Liotta [3], Francesco Giacalone [1] and Michelangelo Gruttadauria [1,*]

[1] Dipartimento Scienze e Tecnologie Biologiche, Chimiche e Farmaceutiche (STEBICEF), Università degli Studi di Palermo, Viale delle Scienze, Ed. 17, 90128 Palermo, Italy; benedetto.taormina@you.unipa.it (B.T.), francesco.giacalone@unipa.it (F.G.)
[2]. Centro Grandi Apparecchiature-ATeN Center, Università degli Studi di Palermo, Via F. Marini 14, 90128 Palermo, Italy; alberto.spinella@unipa.it (A.S.)
[3] Istituto per lo Studio dei Materiali Nanostrutturati ISMN-CNR, Via Ugo La Malfa 153, 90146 Palermo, Italy; leonardafrancesca.liotta@cnr.it (L.F.L.)
* Correspondence: vincenzo.campisciano@unipa.it (V.C.); michelangelo.gruttadauria@unipa.it (M.G.)

Table of Contents:

Structure of compounds and materials **1-4**. **S2**

Figure S1. ^{1}H NMR (300 MHz, DMSO-d$_6$) of compound **1**. **S3**

Figure S2. ^{13}C NMR (75 MHz, DMSO-d$_6$) of compound **1**. **S3**

Figure S3. ^{13}C CP-MAS NMR of material **2**. **S3**

Figure S4. ^{13}C CP-MAS NMR of material **3**. **S4**

Figure S5. TGA graph of materials **2** and **3**. **S4**

Figure S6. N_2-adsorption/desorption isotherms of material **2**. **S4**

Figure S7. N_2-adsorption/desorption isotherms of material **3**. **S5**

Figure S8. ^{1}H NMR (300 MHz, DMSO-d$_6$) of compound **4**. **S5**

Figure S9. ^{13}C NMR (75 MHz, DMSO-d$_6$) of compound **4**. **S5**

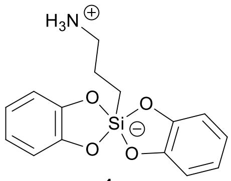

1

3-ammoniumpropylbis(catecholato)silicate **1**.

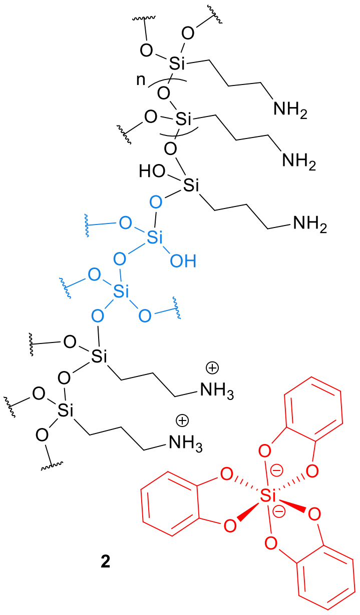

2

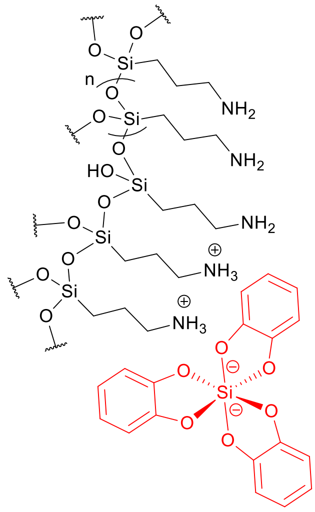

3

Proposed structure for materials **2** and **3**. Material **A** has a structure similar to **2**.

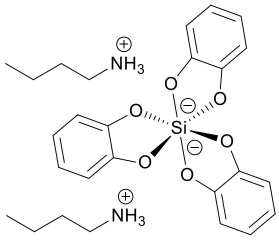

4

Bis(butylammonium) tris(catecholato)silicate **4**.

Structure of compounds and materials **1-4**.

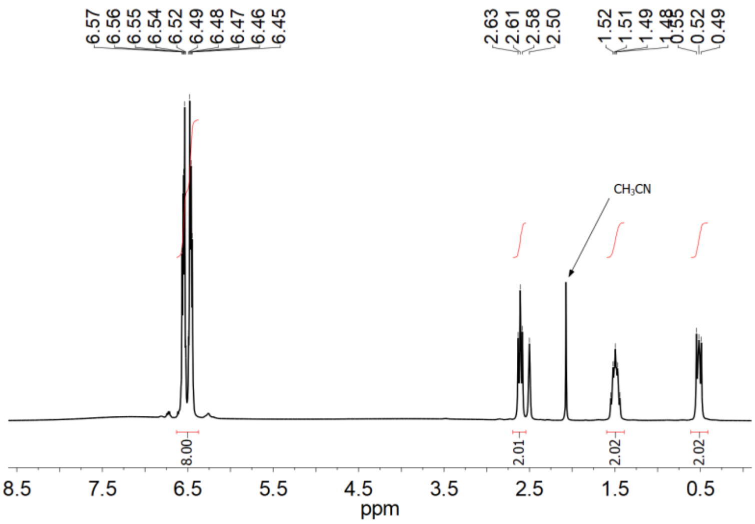

Figure S1. ^{1}H NMR (300 MHz, DMSO-d$_6$) of compound **1**.

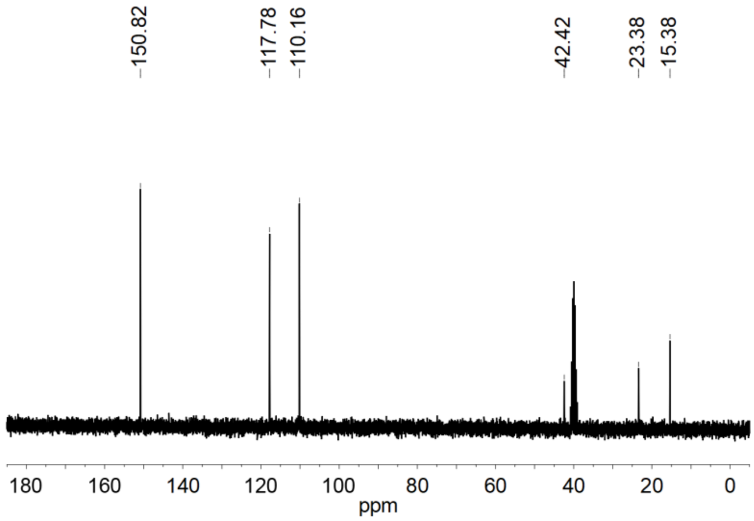

Figure S2. ^{13}C NMR (75 MHz, DMSO-d$_6$) of compound **1**.

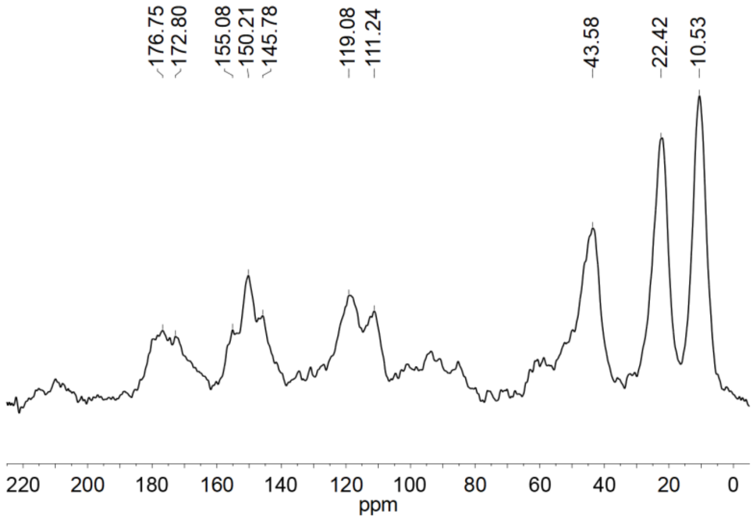

Figure S3. ^{13}C CP-MAS NMR of material **2**.

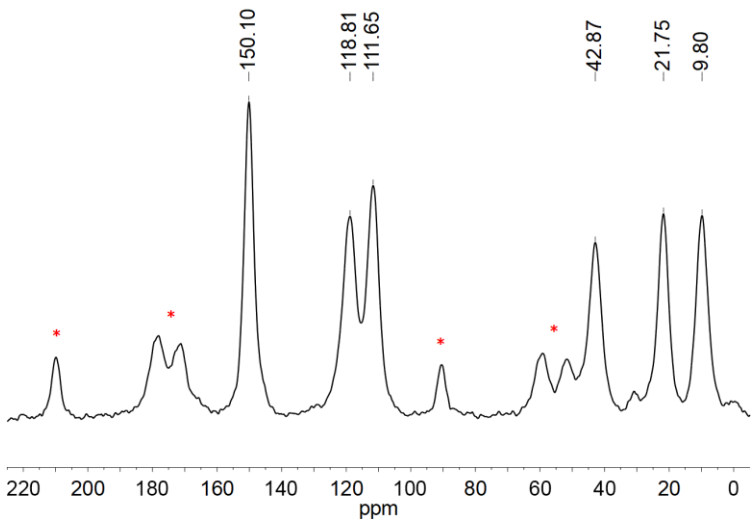

Figure S4. ^{13}C CP-MAS NMR of material **3**.

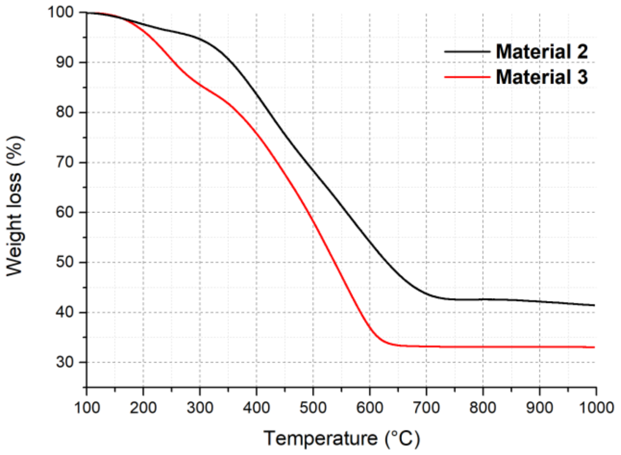

Figure S5. TGA graph of materials **2** and **3**.

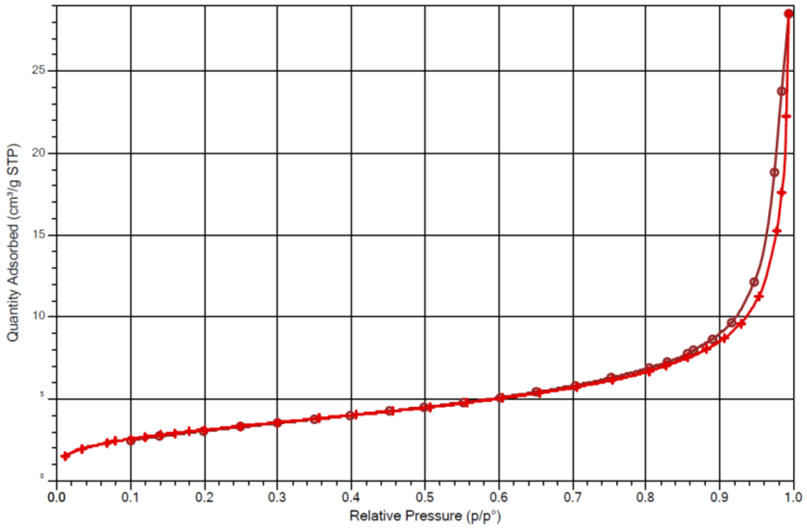

Figure S6. N_2-adsorption/desorption isotherms of material **2**.

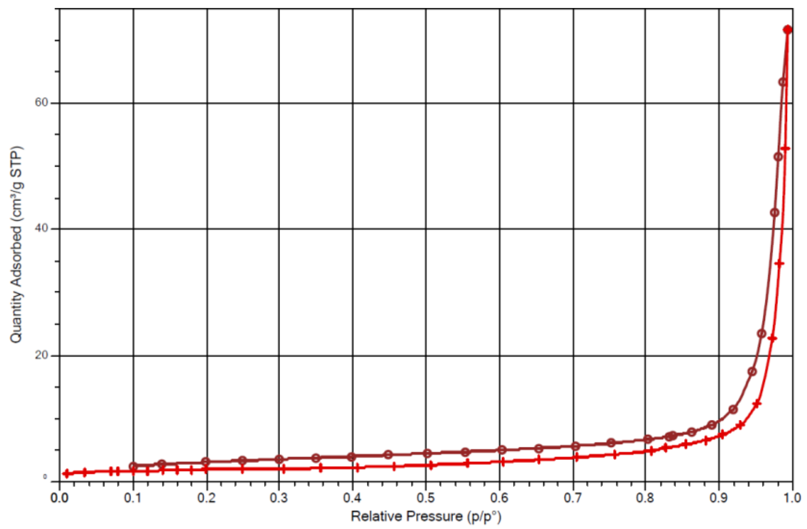

Figure S7. N_2-adsorption/desorption isotherms of material **3**.

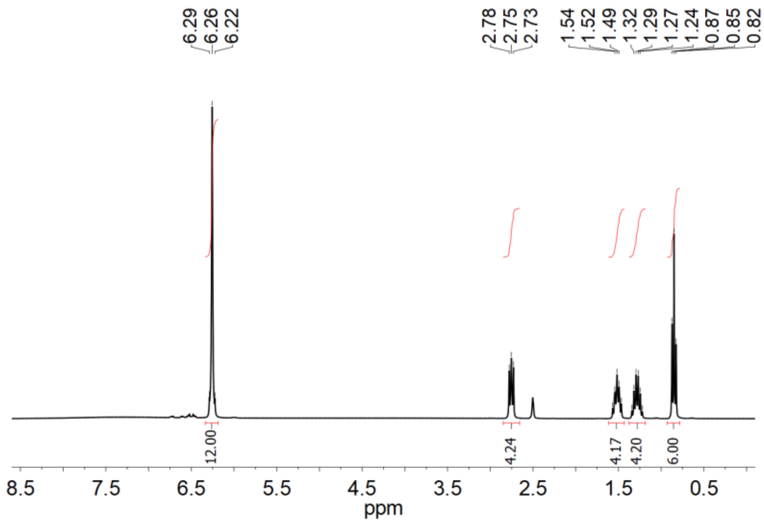

Figure S8. ^{1}H NMR (300 MHz, DMSO-d_6) of compound **4**.

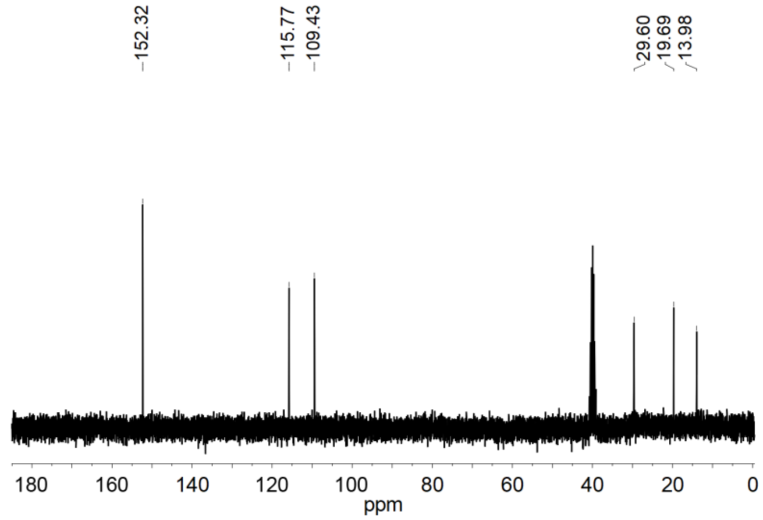

Figure S9. ^{13}C NMR (75 MHz, DMSO-d_6) of compound **4**.
